# Supplementary material for: Patterns of Intron Gain and Loss in Fungi
Source: PLoS Biol. 2004 Nov 30;2(12):e422. doi: 10.1371/journal.pbio.0020422 (PMC532390; doi:10.1371/journal.pbio.0020422)
Supplement: Table S1 — Also available at http://genes.mit.edu/NielsenEtAl/. (4.3 MB ZIP). [file pbio.0020422.st001.zip › NielsenEtAl/html/830.html]

AN2289.1.NCU00450.1.MG06438.1.FG06890.1


```
 CLUSTAL W (1.82) Multiple Sequence Alignments - Introns Inserted


Sequence 1: NCU00450.1	637 aa
Sequence 2: MG06438.1	901 aa
Sequence 3: FG06890.1	647 aa
Sequence 4: AN2289.1	635 aa
Alignment Length: 979 aa
Number Identitical Residues: 196 aa
Alignment Score (without introns) 11554


MG06438.1 	-MPPSSAEPP--AASAANAARHVSIDEP--RRD--------GRKD--PNQNEQSPLLSPQ
NCU00450.1	-MTSSLAEHPDRARPAGNEGHQQQSEQPKSRREEPASRLSTGRRDGASEDTNQQFLVGEQ
FG06890.1 	-----MGVPS--EREAGDGPPASSASNP--RRT---------------PGNQSYARVAAQ
AN2289.1  	MAATDR------------------------------------------AVNETSPLVGAR
          	  ...                                             .:    :. :

MG06438.1 	DDEVDSLMARRESAIEDDDDEIDPFETTKSVWYLILLTISIGG~LQIAWSVELSNGS~PY
NCU00450.1	SPLLSPVDNQDGSSSDQGADSADEFQTTKSVWYMILLTISIGG~LQIAWSVEMSNGS~PY
FG06890.1 	GTDEQSPLLRHDADTDNAHGEHDDSQTTKSIWYLLLLTISIGG~LQIAWSVELSNGS~PY
AN2289.1  	TTDDADVDSMTFVN-GSGNEAPHDMPETKTSWYLFILTLSIGG2LQVVWSVELSSGS0PY
          	                .     .    **: **:::**:**** **:.****:*.** **

MG06438.1 	LLSLGLSKSLMALVWIAGPLSGTLVQPYVGMLSDNCRISWGKRKPFMLVGAAATITSLLF
NCU00450.1	LLSLGISKSLMALVWIAGPLSGTLVQPYVGMMSDNCRIRWGKRKPFMLGGAAATILSLMF
FG06890.1 	LLSLGLSKSLMALVWIAGPLTGTLVQPYVGMLSDNCRMPMGKRKPFMIGGSVATILSLLF
AN2289.1  	LLSLGMSKALLAFVWIAGPITGTLVQPYIGIRSDNCRISWGKRKPFMIVGGAVTIATLLG
          	*****:**:*:*:******::*******:*: *****:  *******: *...** :*: 

MG06438.1 	LAWTREIVSGILGLFGAPADSDGVKTTIIVVAVLWVYILDFAINTE1AANSMASRITGVG
NCU00450.1	LAWTREIVTGILGLFGADPQSESVKLCIICTAVLWIYILDFAINTV1QAAIRAFIVDCAP
FG06890.1 	LAWAKEIVACASNIIGFDPESQGVKTTTIVVAVVGVYVLDFAINTV~QASIRAFIVDCAP
AN2289.1  	LAWVKEFVGGFLVLFGGDPKSEGANIVIGIVATMMMWCLDFAINTV1QAGIRCFIVDNAP
          	***.:*:*     ::*  ..*:..:     .*.: :: *******   *   .  :  . 

MG06438.1 	NI------------------VGYVAGFVNLPKIAWWLGKTQF~QDLCAIAS~IALGVTVI
NCU00450.1	-------------------------------------THQQE1MANAMASS2LALGTTVL
FG06890.1 	AHQQESANAMASRITGFGNIVGYIAGYVDLTRHLGFLGKTQF~QILCAIAC~VALALTVF
AN2289.1  	AHQQESANAWASRMTGVGNIIGYIFGYINLPKLFPYLGNTRF~QILCALAS~ISLAVTLL
          	  ...::.: ::  :. ..  .   .  . .       : :     .  :. ::*. *::

MG06438.1 	ITCILIPERDPRLEGPPPRDQPGVLSFFTKIFTSIKRLPPVTKRVCQVQFCAWVGFFPML
NCU00450.1	MTCLFIKERDPRLEGPPAKDKPGVVAFFKKIFTSIKRLPPQTKKVCQVQFCAWIGFFPML
FG06890.1 	VSTALIKERDPRLDGPAKKQEHGVISFFLTIFKSIKRLPPQIKRVCEVQFCAWVGFFPLL
AN2289.1  	ISCSYIQERDPRLDGPPPSGSMGVVGFFKQVFKSIKHLPPQAAKVCEVQIAAWIAWFPFL
          	::   * ******:**.   . **:.**  :*.***:***   :**:**:.**:.:**:*

MG06438.1 	FYTSAYIGEIYVQPFLRNNPNMTPKELDELYERATRVGTFALLIYSITSLSTNVFLPFFI
NCU00450.1	FYTSSYIGEIYAEPYLEENPNMTDKELDDLYERATQVGTFALLIFAITSLATNIFLPFFI
FG06890.1 	FYTSSYIGEIYVEPYLEANPHMPPEQLNKLYEQATRIGTFALLINSVVSLLTNVFLPFFI
AN2289.1  	FYATTYIGQLYVNPIFEKNPGLSDSDIDKAWEDATRVGTLALLIYALVSFVANITLPIFV
          	**:::***::*.:* :. ** :. .:::. :* **::**:**** ::.*: :*: **:*:

MG06438.1 	QPTYDAAP--------------------------VVSADAPGEGPSVLANAKNANGSSRS
NCU00450.1	APTYDQS---------------------------MVTAVAPGEAPAVVIKDYEPE--QRS
FG06890.1 	APTYDSQ----------------------------PVNNVPGETPAAGHYDEDED-DKPS
AN2289.1  	VPTYKPVLSSDSSEVDIRQEAEPCLRVERQSSSDIEVGLVAELHPEVLRDKARGSAESKS
          	 ***.   ::.::. .  ..:...   . .:::.     ..   * .       .... *

MG06438.1 	WLDRLVIPGFTLRRAWMFSHILFAFSMGCTVLVESVEAATVLIGLVGITWALTLWAPWSL
NCU00450.1	WTRHLIIPGFTLRRAWMFSQILFTGCMLCTVFVRTVTAATVLIGLVGITWALTLWAPWAI
FG06890.1 	WLDKLAIPGLTLKRAWFLSLLLFAGCMFATLFVRTVKAATVLIGLVGITWAMTLWAPWAI
AN2289.1  	WSSKLQIPGLTLRRTWLLSQILFALCMFSTFFITSVTAATVVVGLIGIPWALSLWAPFAL
          	*  :* ***:**:*:*::* :**: .* .*.:: :* ****::**:**.**::****:::

MG06438.1 	ISS1EISRRDAVLRAQQRRR---GLTLDQPYDEDETPSSETSSGQLEVERDEQVAD----
NCU00450.1	ISA~EISQRDEERRSQQQR-----LSPSRLDNLDGYSSDGNQDSDLGKD-DEEAAD----
FG06890.1 	ISA~EISRRDALIRAQRTR----------------QATARANNGGIRGDEEEEEID----
AN2289.1  	ISA~EVATLEEQRRSRRQRSELTTFDEQMSPQIDRDAADDNDDDDDDDDNDDNDAETGSS
          	**: *::  :   *::: * . :    .   . .  .:   ...    : :::  ::.::

MG06438.1 	------------QAGIILGIHNMAIAAPQIIATVGSSIIFKFFQKPRGTPGDRSISIVMA
NCU00450.1	------------QAGVILGIHNMAIAAPQIIATVASSIIFRLFQKPRGTPGDHSIAIVLA
FG06890.1 	------------QAGVILGIHNMAIAAPQIIATVGSSVIFKLWQKPRGTPGDHSISIVFA
AN2289.1  	RSPKTPGDENSAQAGIVLGLHNVAVSAPQVLSSLICSAIFRASQKPRGEPYDESVGWVLR
          	 :..:.....::***::**:**:*::***::::: .* **:  ***** * *.*:. *: 

MG06438.1 	LGGITVLGAAWLVHRIDDDVAVPADELSGIMEAGASDGILTGGPPRPSLSQRTSFSSTRP
NCU00450.1	LGGITVLISALFIHRIRDEPTVTYDEISAVEEG--------DAAPRPNVHSRS-------
FG06890.1 	LGGICVLVSSFFVAKIKDDAAMPADVMIEAEEGQSG---AAEVVERRPLNRRK-------
AN2289.1  	FGGCAALLAAWLTSRLSEG------------------------TPRKR------------
          	:**  .* :: :  :: :                           *              

MG06438.1 	SFSRSRNQSTESLSRYSQERAAL0ICKGEPAAPGFTQAKVPLLRAAGKPMSSITDATVLI
NCU00450.1	-------RSFDQPPRASLERATL~VR----------------------------------
FG06890.1 	-------STRDQLPRATQARANL~TR----------------------------------
AN2289.1  	-----------------------~------------------------------------
          	                                                            

MG06438.1 	GTQTEHV1LPIRSPPARNNAHQQSSYPRPRRPPILSSRAVGRAVQRPKTAQDLNKWSWAN
NCU00450.1	-------~---------NKSFGGMEY----------------------------------
FG06890.1 	-------~---------NKSFGGAEMY---------------------------------
AN2289.1  	-------~----------------------------------------------------
          	                                                            

MG06438.1 	QAGPYQYYIHGSGSVDKYISLSPGYKNPNETASARGARFALDGAVFWIGQNMRRTELISQ
NCU00450.1	------------------------------------------------------------
FG06890.1 	------------------------------------------------------------
AN2289.1  	------------------------------------------------------------
          	                                                            

MG06438.1 	TTAGI0HFTEMTYGWISGEQGTSNKNLQWMVGQRSLWKTEWKPDVWHNVAYEI0DFGART
NCU00450.1	-----~-----------------------------------------------~------
FG06890.1 	-----~-----------------------------------------------~------
AN2289.1  	-----~-----------------------------------------------~------
          	                                                            

MG06438.1 	VGFWHSEGGTPLERKFAPV1SESTSSNGAD
NCU00450.1	-------------------~----------
FG06890.1 	-------------------~----------
AN2289.1  	-------------------~----------
          	
```
